# Linear Fidelity in Quantification of Anti-Viral CD8^+^ T Cells

**DOI:** 10.1371/journal.pone.0039533

**Published:** 2012-06-20

**Authors:** Inge E. A. Flesch, Natasha A. Hollett, Yik Chun Wong, David C. Tscharke

**Affiliations:** Division of Biomedical Science and Biochemistry, Research School of Biology, The Australian National University, Canberra, Australia; The University of Adelaide, Australia

## Abstract

Enumeration of anti-viral CD8^+^ T cells to make comparisons between mice, viruses and vaccines is a frequently used approach, but controversy persists as to the most appropriate methods. Use of peptide-MHC tetramers (or variants) and intracellular staining for cytokines, in particular IFNγ, after a short *ex vivo* stimulation are now common, as are a variety of cytotoxicity assays, but few direct comparisons have been made. It has been argued that use of tetramers leads to the counting of non-functional T cells and that measurement of single cytokines will fail to identify cells with alternative functions. Further, the linear range of these methods has not been tested and this is required to give confidence that relative quantifications can be compared across samples. Here we show for two acute virus infections and CD8^+^ T cells activated *in vitro* that DimerX (a tetramer variant) and intracellular staining for IFNγ, alone or in combination with CD107 to detect degranulation, gave comparable results at the peak of the response. Importantly, these methods were highly linear over nearly two orders of magnitude. In contrast, *in vitro* and *in vivo* assays for cytotoxicity were not linear, suffering from high background killing, plateaus in maximal killing and substantial underestimation of differences in magnitude of responses.

## Introduction

CD8^+^ T cells play a crucial role in anti-viral immunity [1], [2]. Their main functions are the elimination of infected cells by cytotoxicity and production of a range of cytokines after activation through their T cell receptor [3], [4]. Accurate methods to quantify CD8^+^ T cells are fundamental tools in viral immunology. The earliest method to measure CD8^+^ T cell effector activity was the chromium (^51^Cr) release assay which indirectly measures the viability of ^51^Cr-labeled target cells after incubation with effector T cells [5]. This method has long been considered to be poorly quantitative and so was combined with the tedious and time consuming process of limiting dilution in more rigorous studies [1]. Never-the-less comparisons of traditional cytotoxicity backed with statistical analyses, for example to support the superiority of vaccine candidates, remain commonly published. Cytotoxicity assays that use fluorescent dyes and flow cytometry are now becoming more common and also allow a variant of this assay to be done *in vivo*. Using fluorescent dyes such as carboxyfluorescein succinimidyl ester (CFSE), target cell populations loaded with different antigenic peptides can be labeled with the dye at different fluorescence intensities and the cytotoxic activity of CD8^+^ T cells towards multiple target populations can then be assessed in the same assay [6], [7]. An advantage of this assay is that it measures survival of targets rather than an indirect measure of cell death as in the release of ^51^Cr. However these assays remain limited to detecting the killing of targets, rather than the CD8^+^ T cells themselves.

The use of tetrameric MHC/peptide complexes (tetramers) revolutionized the field, allowing precise quantification of epitope-specific CD8^+^ T cells and showing that these are far more frequent (at least 10-fold) than suggested by the previous assays [8]. In addition, other markers of activation, e.g. CD62L, CD44 and granzyme B, can be analyzed in combination with tetramers. However, tetramers and the various variants of this technology alone do not demonstrate functional capability. There are two main methods for detecting the production of cytokines after a brief *ex vivo* stimulation: enzyme linked immunospot assay (ELISpot) and intracellular staining with antibodies and flow cytometric analysis (ICS) [9], [10], [11]. The most commonly detected cytokine is IFNγ for both assays. This choice is supported by evidence that cytokine production by anti-viral CD8^+^ T cells is in a hierarchy where IFNγ is made by most cells, followed by a fraction that also make TNFα and then others that make IL-2 as third effector [12]. Therefore use of IFNγ as a marker should detect the greatest number of virus-specific CD8^+^ T cells with a simple protocol for ELISpot or ICS. Here we focus on the ICS approach and refer to the whole assay, including stimulation, as IFNγ-ICS. IFNγ-ICS can be combined with detection of surface CD107a/b to demonstrate degranulation, which is required for cytotoxic function, during stimulation [13], [14]. This allows at least one aspect of each of the two major CD8^+^ T cell functions to be combined with direct detection of CD8^+^ T cells. However, two caveats remain: some populations of CD8^+^ T cells may respond by making cytokines other than IFNγ and the ability to degranulate is only one of the requisites for cytotoxic ability [15]. More recently an emphasis on polyfunctionality (the ability to make several cytokines and exert cytotoxicity) has been introduced as it is clear that CD8^+^ T cell quality is important, as well as quantity [16]. However, it remains important to quantify the denominator for any such studies and this will remain the total number of CD8^+^ T cells capable of responding to a given viral specificity.

A central issue persists: how well do common assays that are used to compare the size of CD8^+^ T cell responses stack up? Anecdotal evidence and common opinion in the field seems to be that IFNγ-ICS fails to account for all anti-viral CD8^+^ T cells. However direct comparisons of tetramers and IFNγ production at the single cell level have given conflicting results [17]. Use of IFNγ-based assays have been reported to detect fewer [18], more [19], or similar numbers [20], [21] of antigen-specific CD8^+^ T cells than tetramers. Inclusion of degranulation as an extra marker has not been examined and rigorous tests of the linear range of each are also lacking. Simple staining with tetramers or similar reagents followed by flow cytometry would be expected to give linear results. However, where cells require activation by stimulation, as in IFNγ-ICS it is possible that the dynamics of culture might introduce unexpected threshold effects.

In this study, we have addressed these issues using vaccinia virus (VACV) and herpes simplex virus type 1 (HSV-1) infections of mice and *in vitro* activated OT-I CD8^+^ T cells. In our first approach we took advantage of recombinant viruses lacking a dominant CD8^+^ T cell epitope and titrated splenocytes from mice infected with a wild type virus into those from mice infected with the epitope-deletion mutant. This allowed us to simulate real samples with differing, but predictable levels CD8^+^ T cells of a known specificity diluted by splenocytes from similarly infected mice. The methods chosen for comparison were a) the DimerX variant of tetramer technology [22], [23], b) IFNγ-ICS, c) IFNγ-ICS combined with CD107 mobilization (IFNγ-ICS/CD107), and d) a CFSE-based *in vitro* cytotoxicity assay. In a second approach, we constructed different levels of epitope-specific CD8^+^ T cells *in vivo* by activating OT-I cells *in vitro* and transferring known numbers of these into mice. This enabled the inclusion of an *in vivo* cytotoxicity assay in our comparisons.

## Results

### Reproducibility of IFNγ-ICS

**Figure 1:**
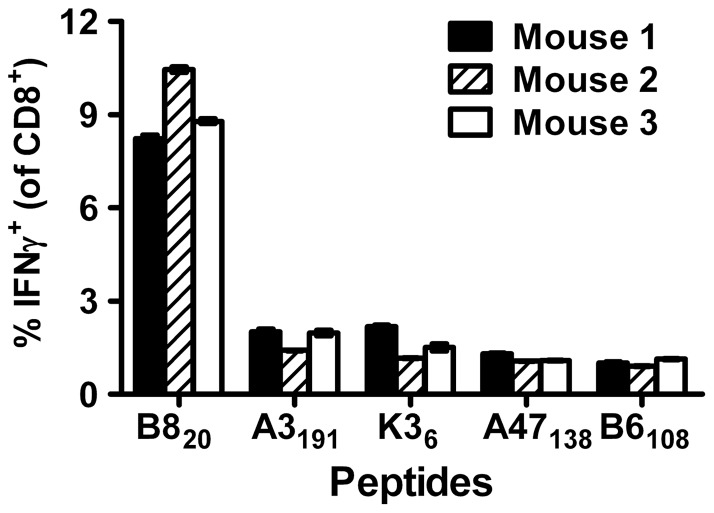
Reproducibility of IFNγ-ICS. Three mice were infected i.p. with VACV WR and after seven days CD8^+^ T cell responses to a set of peptides (as shown on the figure) were measured by IFNγ-ICS assays done in quadruplicate for each peptide and each mouse. Backgrounds determined using irrelevant peptides were subtracted from the values presented. Data are mean ± SEM. Reproducibility was similar in a second experiment in which spleens were pooled from two infected mice and responses to the set of peptides above assayed with 10 replicates.

IFNγ-ICS often shows substantial variance (shown by quite large errors for some measurements) where CD8^+^ T cells are enumerated using multiple mice as replicates [22], [24], [25]. We wondered if this was a true reflection of the size of responses in these mice or was generated by errors associated with the assay itself. Therefore before attempting direct comparisons between methods, we wanted to test the reproducibility of IFNγ-ICS, one method that relies both upon brief *in vitro* culture and antibody staining for flow cytometric analysis. A group of three mice was infected with VACV and seven days later, which is the peak of the response, CD8^+^ T cell responses to five epitopes were measured by IFNγ-ICS, each epitope for each mouse being assayed in quadruplicate (Figure 1). We were surprised by the reproducibility of the IFNγ-ICS assay, as shown by the very small error, and also by the extent of differences between individual mice. Indeed, a one way ANOVA comparing all three mice found statistically significant differences across the mice for CD8^+^ T cell responses to all epitopes and a post-test found differences for 13 of 15 possible pair-wise comparisons. We concluded that the IFNγ-ICS assay is highly reproducible and that substantial differences can exist in responses of different mice to the same epitopes in the same infection.

### IFNγ-ICS, DimerX and IFNγ-ICS/CD107 are highly linear and give comparable estimates of VACV-specific CD8^+^ T cells

To directly compare numbers of CD8^+^ T cells detected and the linearity of results produced by DimerX, IFNγ-ICS and IFNγ-ICS/CD107 mobilization, splenocytes from VACV-infected mice were serially diluted into splenocytes from VACV ΔB8R [26] -infected mice. This created a series of samples in which B8_20_-specific CD8^+^ T cells are diluted in two-fold steps from their usual level (around 6–8% of CD8^+^ T cells) to 64-fold less than this amount. As a control, A3_270_-specific CD8^+^ T cells were quantified in parallel. In all assays, the CD8^+^ T cell response to B8_20_ was expected to decrease, while the response to A3_270_ should remain the same because the total number of splenocytes in each well was kept constant.

**Figure 2:**
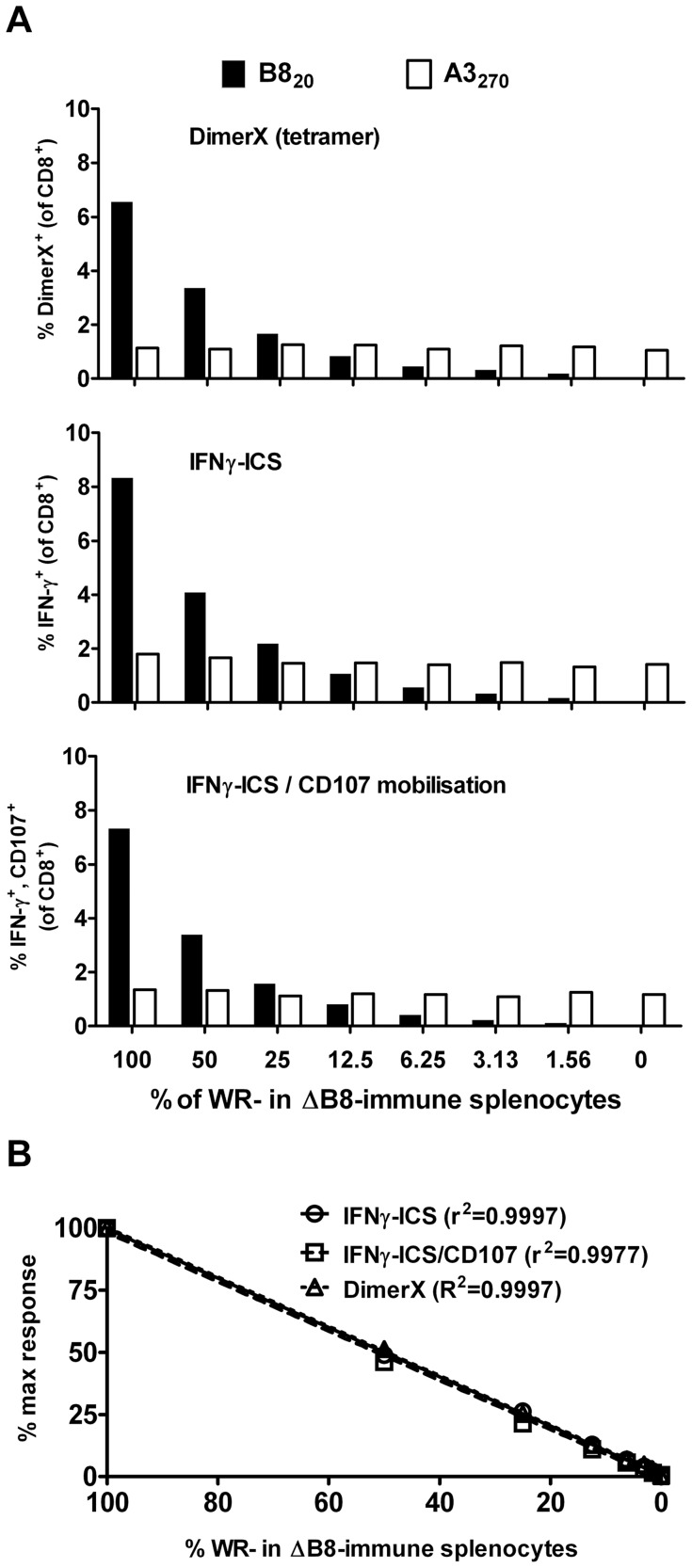
Direct quantification of VACV-specific CD8^+^ T cells. Splenocytes from mice infected with VACV-WR were serially diluted in splenocytes from mice infected with VACV-ΔB8R-WR and CD8^+^ T cell responses to the peptides B8_20_ and A3_270_ were measured using three methods. (A) Data from DimerX, IFNγ-ICS and IFNγ-ICS/CD107 methods as indicated on each graph. Backgrounds determined using irrelevant peptides were subtracted from the values presented. (B) Linear regression analysis and r^2^ statistics for quantification of B8_20_–specific CD8^+^ T cells by each method. Data are representative of two independent experiments.

All methods gave similar results, but with DimerX consistently giving the lowest estimate of the frequency of epitope-specific CD8^+^ T cell responses and IFNγ-ICS the highest (Figure 2A). This was seen for B8_20_, which varied across the dilution series as expected, and for A3_270_, which stayed constant. However, the difference between these methods was within the range that responses were shown to vary between mice above. We then used linear regression to analyze data from the titration of B8_20_ responses to determine if each method accurately reflected the expected dilutions (Figure 2B). For all three methods, the results obtained demonstrated extremely high fidelity to the expected result, with r^2^ values very close to one, the lowest being 0.9977 for IFNγ-ICS combined with CD107 mobilization. In a second experiment, the three methods gave even more similar estimates of the size of the B8_20_-specific response (DimerX, 6.42%; IFNγ-ICS, 6.75%; IFNγ-ICS/CD107, 6.42%), being within the range of replicates in the IFNγ-ICS assay shown in Figure 1. Again r^2^ values for these methods were close to one (DimerX, 0.9771; IFNγ-ICS, 0.9910; IFNγ-ICS/CD107, 0.9758).

### Cytotoxicity assays reflect actual differences in CD8^+^ T cell responses poorly

**Figure 3:**
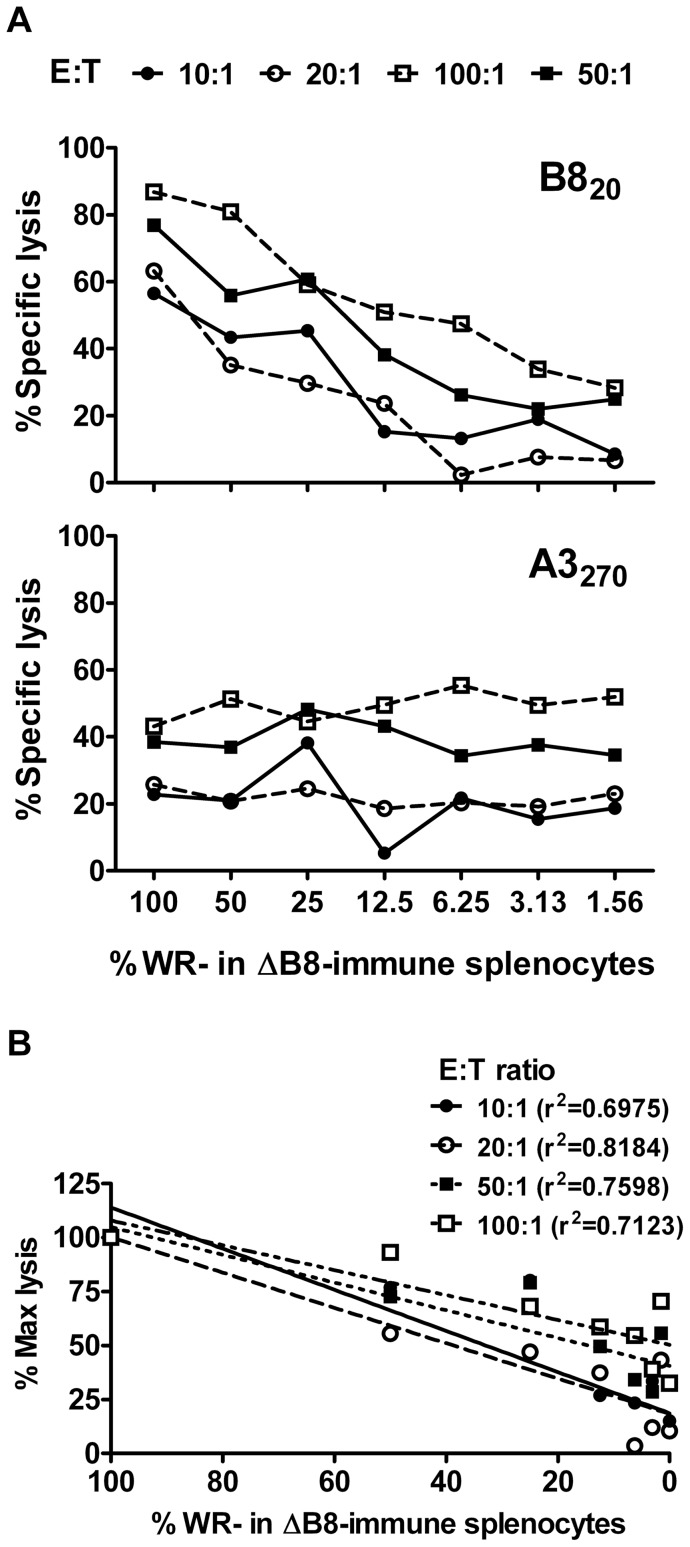
Measuring in vitro cytotoxicity of VACV-specific CD8^+^ T cells. The same set of serial dilutions of splenocytes from VACV WR and VACV ΔB8R mice as made for the experiments in figure 2 were used as a source of effectors in an *in vitro* cytotoxicity assay. Target cells were RMA cells coated with B8_20_ or A3_270_ and these were incubated with effectors at the E:T ratios shown. (A) The percent loss of B8_20_ or A3_270_ (as shown on graphs) coated cells as compared with non-peptide-coated control targets. (B) Linear regression analysis and r^2^ statistics derived from the B8_20_–specific killing data shown in panel A. Data are representative of two independent experiments.

*In vitro* cytotoxicity assays were done using the same dilution series of splenocytes as described above. The assay employed was based on loss of fluorescently labeled target cells coated with relevant peptides compared with those coated with an irrelevant peptide as detected by flow cytometry. The use of two dyes allowed the simultaneous measurement of B8_20_- and A3_270_-specific killing in the same well. Further as is typical in cytotoxicity assays, four E:T ratios were tested. As an overall impression, the level of B8_20_-specific killing did fall for each E:T ratio as the WR-immune splenocytes were diluted with ΔB8-immune splenocytes, but this was not true for each two-fold step (Figure 3A). Over the full 64-fold dilution series, the drop in killing was approximately 3-fold, suggesting that the cytotoxicity assay greatly underestimates differences in actual responses. A3_270_-specific killing across the same set of samples suggested similar killing across the samples, as expected but again some individual wells gave irregular results. To formalize the comparison of measured killing to actual CD8^+^ T cell numbers for the B8_20­_–specific response, regression analysis was done for each E:T ratio (Figure 3B). The r^2^ values for the various E:T ratios ranged from 0.6975 to 0.8184, all indicating that the cytotoxicity assay fails to render differences in CD8^+^ T cell responses in a linear fashion.

### IFNγ-ICS, DimerX and IFNγ-ICS/CD107 are highly linear and give comparable estimates of HSV-specific CD8+ T cells

**Figure 4:**
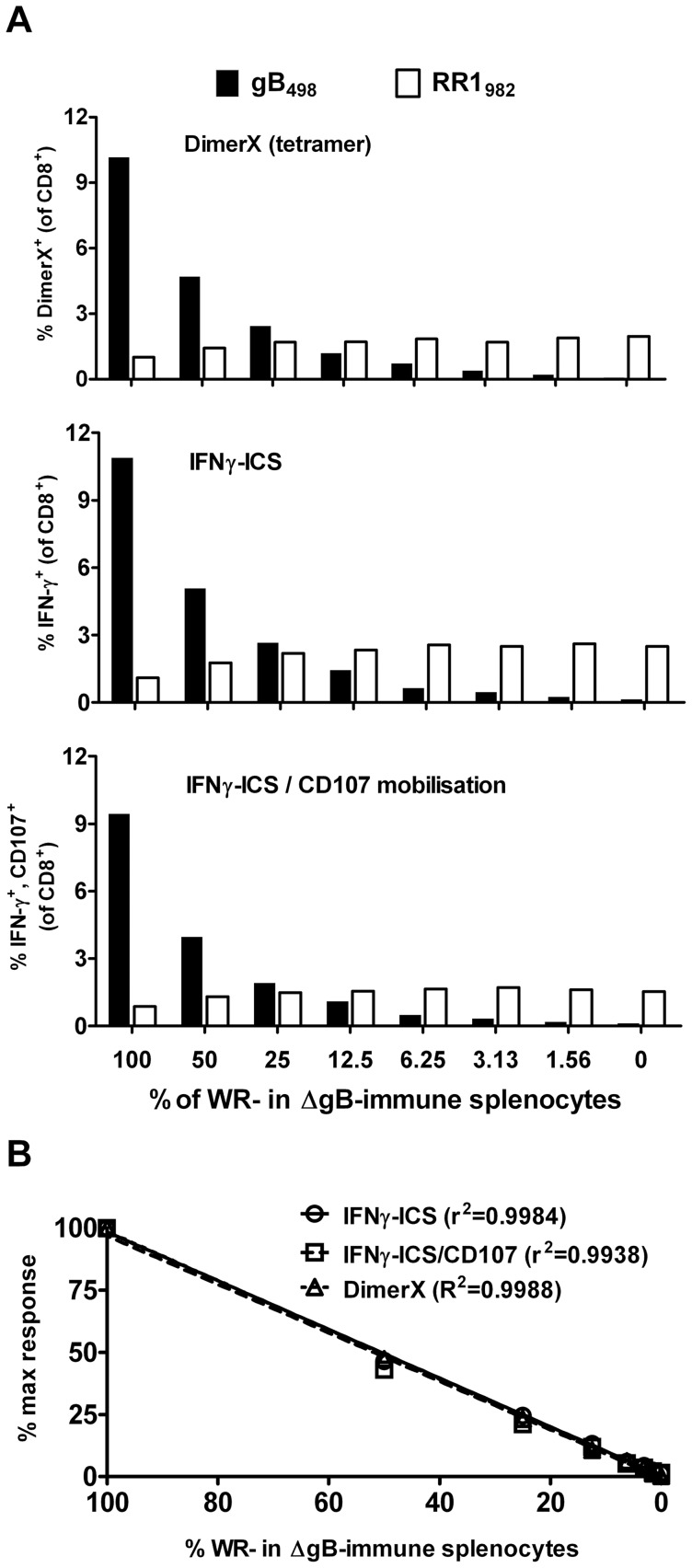
Direct quantification of HSV-specific CD8^+^ T cells. Splenocytes from mice infected with HSV KOS were serially diluted in splenocytes from mice infected with HSV K.L8A and CD8^+^ T cell responses to the peptides gB_498_ and RR1_982_ were measured using three methods. (A) Data from DimerX, IFNγ-ICS and IFNγ-ICS/CD107 methods as indicated on each graph. Backgrounds determined using irrelevant peptides were subtracted from the values presented. (B) Linear regression analysis and r^2^ statistics for quantification of gB_498_–specific CD8^+^ T cells by each method.

Next we wanted to extend our analyses to another acute virus infection, to see if the equivalence of methods was a peculiar feature of VACV infections. To do this we infected the flanks of mice with HSV KOS and KOS K.L8A [27] and took spleens for analysis seven days later. Splenocytes from HSV KOS infected mice were diluted with splenocytes from KOS K.L8A infected mice to make a similar set of dilutions as was done for VACV (above). Reponses to the HSV epitopes HSV gB_498_ (expected to be diluted across the series) and RR1_982_ (constant) were measured using DimerX, IFNγ-ICS and IFNγ-ICS/CD107 methods (Figure 4A). As was seen for the anti-VACV responses, all three methods gave similar estimates of HSV-specific CD8^+^ T cell responses. Further the linear fidelity of each of these methods was also confirmed (Figure 4B). In contrast to the VACV experiment, we noticed that the responses to the RR1_982_ epitope also changed across the dilution series. The response to this peptide was lowest in samples with splenocytes only from KOS-infected mice and highest where they were all from KOS K.L8A-infected mice. This was most likely due to immunodomination by gB_498_ in mice infected with KOS and indeed the increase of response to RR1_982_ as KOS-immune splenocytes were diluted in K.L8A-immune splenocytes was linear.

### In vivo detection of antigen-specific CD8+ T cells by DimerX assay, IFNγ-ICS and a CFSE-based cytotoxicity assay

**Figure 5:**
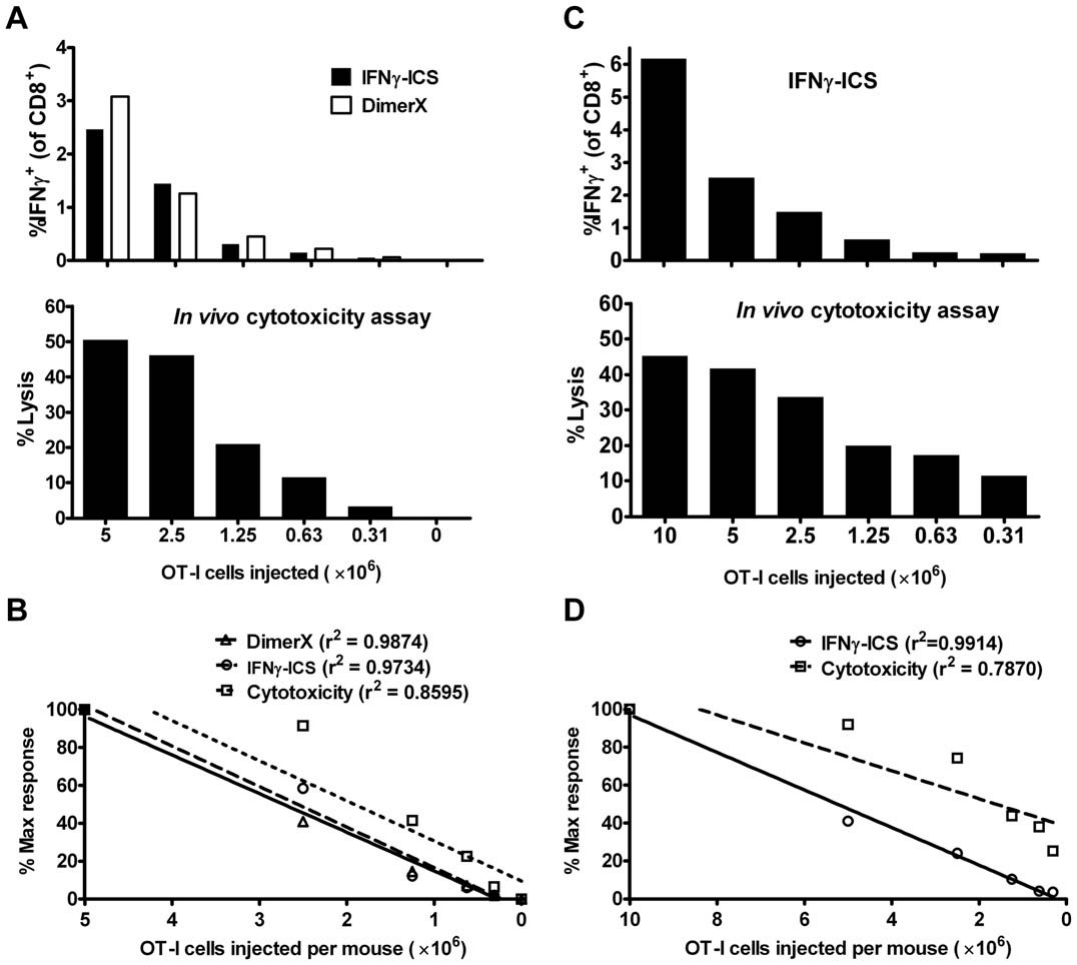
Comparison of DimerX, IFNγ-ICS and an in vivo cytotoxicity assay. OVA_257_-specific CD8^+^ T cells were generated from OT-I mice and increasing numbers were injected i.v. into B6 mice. After 24 hours, naïve B6.SJL splenocytes coated with OVA_257_ (labeled CFSE^high^) mixed with uncoated splenocytes (labeled CFSE^low^) were injected i.v. into the OT-I T cell-treated B6 mice. After 4 hours, splenocytes were prepared and the percentage of OVA_257_-specific CD8^+^ T cells was determined by DimerX and/or IFNγ-ICS and OVA_257_–specific killing measured by comparing recovery of CD45.1^+^ CFSE^high^ and CFSE^low^ cells. Two experiments are shown (A and C) and graphs are labeled with the method used. (B and D) Linear regression analysis and r^2^ statistics for the experiments shown in panels A and C, respectively.

The *in vivo* cytotoxicity assay is now very frequently used, so we included this method in our comparison. This required a gradient of CD8^+^ T cells of known specificity to be generated in a set of mice. To do this we transferred two-fold dilutions of *in vitro*-activated CD8^+^ OT-I T cells into a set of 5 mice. We used a standard CFSE-based *in vivo* cytotoxicity assay, with targets being allowed 4 hours for killing in the mice. The activation of OT-I *in vitro* and the short assay time after transfer of targets eliminates the complication of an endogenous response to SIINFEKL. When spleens were taken to assess killing, we also used the splenocytes to detect OVA_257_-specific CD8^+^ T cells by IFNγ-ICS and DimerX staining. In the first experiment, we started with a maximum of 5×10^6^ CD8^+^ OT-I cells. As seen in other experiments DimerX and IFNγ-ICS again proved highly linear (r^2^ well above 0.95), with these two assays giving similar results considering the whole dilution series (Figure 5A and B). The *in vivo* cytotoxicity assay appeared to have a plateau with less than 10% difference in killing between the mice receiving the two highest doses of OT-I cells, but in mice receiving between 2.5×10^6^ and 3.125×10^5^ cells, the cytotoxicity showed relatively good linear fidelity (r^2^ = 0.9965). To test this apparent plateau, followed by a range of linear response, a second experiment was done, this time starting with 1×10^7^ CD8^+^ OT-I cells (Figure 5C and D). Here only IFNγ-ICS was used for comparison and again this assay was highly linear. In contrast, we confirmed the plateau in killing for high numbers of CD8^+^ OT-I cells, but in this experiment only one step (2.5×10^6^ to 1.25×10^6^ OT-I) approximated the expected two-fold difference and the overall r^2^ was lower for this than the first experiment (r^2^ = 0.7670). Suggesting that the linear range we saw in the first experiment is not a consistent feature of *in vivo* cytotoxicity assays.

## Discussion

Here we present a rigorous comparison of the linear fidelity of several frequently used methods for enumerating anti-viral or vaccine-specific CD8^+^ T cells. The main motivation was our frequent experience of statements about the limitations of various methods being made, but rarely backed by evidence beyond anecdote. The following comments are restricted to the situations that we studied here, namely acute viral infection in immunocompetent mice.

In our first experiment, we were surprised at the reproducibility of the IFNγ-ICS assay, expecting that in other cases where assays for each mouse were done once, variance would be roughly equally derived from real differences between mice and errors associated with the assay. In practice the majority of variance is accounted for by mouse-to-mouse variation. As an additional observation of interest, where responses to the dominant B8_20_ were highest (mouse 2), responses to the less dominant epitopes were reduced. This suggests that immunodomination does play a demonstrable, if small role in setting the peak responses to epitopes in this model, contrary to a recent report [28].

We show clearly here that in anti-VACV and -HSV responses there are very few if any DimerX^+^ CD8^+^ T cells that fail to express an anti-viral function *in vitro* and that similar numbers of CD8^+^ T cells respond to peptide by making IFNγ alone, as make IFNγ and degranulate. This is based on results across multiple experiments and for levels of epitope-specific CD8^+^ T cells spanning nearly two orders of magnitude, which is the typical range seen in most publications. However, some considerations apply to these results. First, the use of DimerX allows a highly similar negative control reagent to be used because these reagents are loaded with a test, or irrelevant peptide and dimerized with the secondary antibody immediately prior to each experiment. Without subtracting values from this negative control DimerX reagent, the results from the tetramers would be higher and a discrepancy between this and the functional methods would be found. With the original tetramer technology, the quality of each reagent can differ between batches of monomer as can the efficiency of tetramerization, which is also done on a batch basis. This means that there is a unique non-specific background associated with each batch of any tetramer that cannot be accurately controlled unless a virus or vaccine lacking the antigen of interest is used and this is rarely practical. Inability to account for background may inflate estimations of epitope-specific CD8^+^ T cells using these reagents. Second, we were careful to use well verified CD8^+^ T cell epitopes for these studies. Where poorly mapped peptides are used, even at the high concentrations typically used for IFNγ-ICS assays, they may fail to trigger all possible responses by all clones of CD8^+^ T cells that are generated by the *bona fide* epitope *in vivo*. This is because the ability to express various functions is related to antigen density [29], [30] and if this threshold is artificially high owing to an incorrectly mapped peptide being used, this may result in apparent functional differences in the responding CD8^+^ T cells. Third, related to the last point, the elicitation of any anti-viral function *ex vivo* triggered by unphysiologically high amounts of peptide cannot be taken to infer that this response occurs *in vivo*. Results gained with IFNγ-ICS or ELISpot assays are often referred to as the ‘IFNγ-response’, but the expression of this cytokine in the anti-viral response will depend on the functional avidity of the CD8^+^ T cells and the level of epitope that is presented on virus infected cells *in vivo* [12]. The latter parameter is at present not known for any epitope from any virus. This together with our data shown here suggest that IFNγ-ICS as used with typically high peptide concentrations (1×10^−7^ M or greater) is a more appropriate method for counting all antigen-specific CD8^+^ T cells than for inferring any functional capacity. Until the amounts of any peptide presented on infected cells *in vivo* are known, even diluting peptides to levels that are closer to the physiological range does not give certainty that the measured function will be manifest in fighting infection. Finally, it cannot be assumed from the above that IFNγ-ICS and DimerX or tetramer staining will always give equivalent results. Examples might include knock-out or mutant mice with defects in aspects of T cell function, the study of pathogens that adversely affect immune function or that dramatically skew responses away from a typical Th1 profile. In addition, we have not tested memory CD8^+^ T cells. However, our data from experiments where OT-I cells were activated *in vitro* and transferred into mice suggest that the data is very likely to be generalizable beyond the VACV and HSV models. So we conclude that these results will hold for the majority of acute anti-viral responses and perhaps other intracellular pathogens.

In contrast to the methods discussed above, both assays of cytotoxicity failed to accurately reflect differences in CD8^+^ T cell numbers. The main deficiency of the *in vitro* assay was that while results roughly correlated with number of effectors, they led to a gross underestimation of differences, with the 64-fold range of CD8^+^ T cell numbers being compressed to less than four-fold difference in cytotoxicity. However, the regularity of the decline of lysis with cell number suggests that if a standard curve could be constructed (as we have done), then a rough estimation of the real difference in response underlying any difference in lysis could be made. This is not possible in most cases so applications of this method used to make quantitative statements should be interpreted with great caution. The *in vivo* assay likewise failed to reflect real differences in CD8^+^ T cells, but in this case a plateau effect was seen for higher numbers of antigen-specific CD8^+^ T cells. It is important to add that the experiment was constructed to assay numbers of OT-I CD8^+^ T cells across a range that typically occurs in anti-viral CD8^+^ T cell responses, so this plateau would be experienced in real-world applications. We used a relatively short assay time (four hours) for the *in vivo* cytotoxicity assay and so where longer times are used, it is likely that the plateau would extend to lower numbers of cytotoxic CD8^+^ T cells. Conversely, shortening the time of the assay might allow better dissection of responses where frequency of effectors is high. It is possible that the time required to achieve a particular level of lysis might be a better correlate of the frequency of effectors, but this would be a very impractical assay and so was not tested here. Our *in vivo* cytotoxicity assay only examined killing of targets in the spleen. At very early times (two days after infection) killing is confined to a single lymph node [31], which implies that it occurs in secondary lymphoid tissue, but by the peak of the CD8^+^ T cell responses it is seen across lymph nodes and spleen and so is probably systemic [31], [32], [33]. This suggests that the spleen is good as a representative organ for measuring cytotoxicity. However, killing is also reflected in some non-lymphoid tissues (lung, liver) [32], [33] and it remains possible that the results at these sites might differ from spleen. Finally, both these assays of cytotoxicity rely on targets coated with unphysiologically high levels of peptide and so they are unable to provide certainty that the lysis measured would translate to killing of infected cells in a real anti-viral response.

In conclusion, we show strong data here to support the reproducibility and linear fidelity of IFNγ-ICS alone or combined with CD107 as a marker for degranulation as well as its similarity to DimerX staining in acute anti-viral CD8^+^ T cell responses. By contrast, assays of cytotoxicity *in vitro* or *in vivo* fail to accurately reflect differences in CD8^+^ T cell responses. These data are helpful to interpret the wealth of published comparisons of CD8^+^ T cell immunogenicity, especially in the field of vaccinology where accurate pre-clinical assessment of different candidates is crucial.

## Materials and Methods

### Viruses and cell lines

VACV strain Western Reserve (VACV WR, ATCC #VR1354) was grown and titrated in cells respectively using standard methods. VACV strain WR was a gift of Bernard Moss (NIH, Bethesda). VACV ΔB8R was a gift from Geoffrey L Smith (University of Cambridge) and has been shown to have equal virulence in mice compared with parental strain, VACV WR [26]. HSV-1 strain KOS and KOS variant K.L8A, which lacks the anchor residue of the dominant gB_498–505_ peptide, but has wild type virulence [27], were a gift from Francis Carbone. All viruses were grown and titrated by standard methods using BHK-21 and BS-C-1 respectively for VACV and Vero cells for HSV-1. Immortalized cell lines BHK-21, BS-C-1 and RMA were maintained in Dulbecco's Modified Eagle medium (DMEM, Invitrogen) with 2 mM L-glutamine and 10% fetal bovine serum (FBS) (D10). Vero cells were grown in Minimal Essential Medium supplemented with 10% FBS, 2 mM L-glutamine, 5 mM HEPES and 50 μM 2-mercaptoethanol (all Invitrogen).

### Synthetic peptides

Lyophilized peptides were purchased from Genscript Corp. (Piscataway, NJ) or Mimotopes (Clayton, Vic Australia). Master stocks of peptides were made at 10 mg/ml in 100% dimethylsulfoxide (DMSO) and stored at −70°C. Before use, peptides were diluted to the required concentrations in serum-free DMEM with L-glutamine (D0). Peptide sequences used were: VACV B8_20-27_, TSYKFESV [34]; VACV A3_270–277_, KSYNYMLL [35]; VACV K3_6–15_, YSLPNAGDVI [34]; VACV A47_138–146_, AAFEFINSL [34]; VACV B6_108–116_, LMYDIINSV [35]; HSV gB_498–505_, SSIEFARL [36]; HSV RR1_982–989_, FAPLFTNL [37]; chicken ovalbumin_257–264_ SIINFEKL [38].

### 5(6)-Carboxyfluorescein-diacetate N-succinimidyl (CFSE)

CFSE was purchased from Sigma-Aldrich. A stock solution was made at 1 mg/ml in DMSO and aliquots were stored at −20°C. Before use, stocks were diluted to required concentrations in D0.

### Ethics Statement

All experiments were done according to Australian NHMRC guidelines contained within the Australian Code of Practice for the Care and Use of Animals for Scientific Purposes and under approvals F-BMB-38.8 and A2011-01 from the Australian National University Animal Ethics and Experimentation Committee.

### Mice and infections

Specific pathogen-free female C57BL/6 mice, C57BL/6.SJL mice and OT-I transgenic [39] mice greater than 8 weeks of age were obtained from Animal Resource Centre (Perth, Australia) or the ANU Bioscience Resource Facility. Mice were infected intraperitoneally (i.p.) with 1×10^6^ plaque forming units (PFU) of VACV in 200 μl PBS. Alternatively mice had left flanks shaved and depilated (Veet cream for sensitive skin, Reckitt Benckiser) before being infected with HSV-1 by tattoo, using a 10RS needle cluster dipped in virus at 1×10^8^ PFU/ml and applied for 10 seconds. This method for HSV inoculation results in similar pathogenesis compared with infection by scarification as originally described [40].

### DimerX reagent and monoclonal antibodies (mAbs)

Recombinant soluble dimeric mouse H-2K^b^:Ig fusion protein and anti-mouse IgG_1_ (clone A85-1) conjugated with phycoerythrin (PE) were purchased from BD Biosciences. Anti-mouse CD8α (clone 53–6.7) conjugated with PE or allophycocyanin (APC), anti-mouse IFNγ (clone XMG.2) conjugated with APC, anti-mouse CD107a (LAMP-1; clone 1D4B) and anti-mouse CD107b (Mac-3; clone M3/84) labeled with fluorescein isothiocyanate (FITC) and anti-mouse CD16/CD32 (Fc-block) (clone 2.4G2) were obtained from BD Biosciences or BioLegend.

### Preparation of and serial dilution splenocytes

Splenocytes were prepared from VACV-WR- and VACV-ΔB8R-infected mice and adjusted to 1×10^7^ cells/ml. To make a series of samples where B8_20_-specific CD8^+^ T cells are diluted, splenocytes from VACV-WR-infected mice were mixed with splenocytes from VACV-ΔB8R-WR-infected mice in a series of steps to achieve the range of 100% to 1.5625%. The same method was used to dilute splenocytes from HSV-1 KOS-infected mice into those from and K.L8A-infected mice. For DimerX, IFNγ-ICS and IFNγ-ICS/CD107 assays, 100 µl of mixed splenocytes were used per well.

### Detection of peptide-specific CD8+ T cells using DimerX reagents

DimerX staining was performed according to the manufacturer's instructions. Briefly, 2 µg of H-2K^b^:Ig fusion protein was incubated overnight at 37°C in PBS with a 40 M excess of a test or irrelevant peptide. Peptide-loaded dimers were then incubated for 1 h at room temperature with PE-conjugated anti-mouse IgG_1_ (clone A85-1). Binding the bivalent secondary antibody turns the DimerX reagents into dimers and as each of these displays two peptide MHC surfaces, the final staining reagent is effectively a peptide-MHC tetramer. Splenocytes (1×10^6^ per sample) were labeled with peptide-loaded dimers and 1/200 anti-CD8-APC for 1 h on ice and washed twice before acquisition on a FACS LSR II (BD Biosciences). Analysis was done using Flowjo software (Tree Star Inc.). Events were gated for live lymphocytes on FSC × SSC followed by CD8^+^ T cells × DimerX^+^ cells. Backgrounds determined by using dimers loaded with irrelevant peptide were subtracted from the values presented for test samples and were generally in the order of 0.5%.

### IFNγ ICS

Splenocytes (100 µl of a 1×10^7^ per ml suspension in D10) were plated in wells of round-bottom 96-well plates. Peptides were added to a final concentration of 10^−7^ M and plates were incubated at 37°C and 5% CO_2_. After 1 h, 5 µg/ml brefeldin A (Sigma) was added, and plates were incubated for another 3 h. Plates were spun at 4°C, medium was removed, and cells were resuspended in 50 µl of 1/150 diluted anti-CD8-PE. After 30 min incubation on ice, cells were washed, resuspended in 50 µl of 1% paraformaldehyde, and incubated at room temperature for 20 min before another two washes and staining with 50 µl of 1/200 diluted anti-IFNγ-APC in PBS with 2% FBS and 0.5% saponin (Sigma) at 4°C overnight. Cells were washed three times before acquisition using a FACS LSR II. Analysis was done using Flowjo software. Events were gated for live lymphocytes on FSC × SSC followed by CD8 × IFNγ. Data was recorded as IFNγ^+^ events as a percentage of total CD8^+^ events. Backgrounds as determined using irrelevant peptides were usually in the order of 0.1% or less and were subtracted from the values presented for test samples.

### Simultaneous staining of cell surface CD107a/b and IFNγ-ICS

Splenocytes (100 µl of a 1×10^7^ per ml suspension in D10) were plated in wells of round-bottom 96-well plates. Fc-block, peptides (10^−7^ M), Golgi-Stop (BD Biosciences) and a 1∶1 mixture of anti-CD107a/b-FITC was added. Plates were incubated at 37°C and 5% CO_2_ for 4 h. Plates were spun at 4°C, medium was removed, and cells were resuspended in 50 µl of 1/150 diluted anti-CD8-PE. After 30 min incubation on ice, cells were washed, resuspended in 50 µl of 1% paraformaldehyde, and incubated at room temperature for 20 min before another two washes and staining with 50 µl of 1/200 diluted anti-IFNγ-APC in PBS with 2% FBS and 0.5% saponin overnight at 4°C. Cells were washed three times before acquisition using a FACS LSR II. Analysis was done using Flowjo software. Events were gated for live lymphocytes on FSC × SSC followed by CD8^+^ T cells using CD8 × SSC and displayed as CD107a/b^+^ × IFNγ^+^. Data was recorded as CD107a/b^+^, IFNγ^+^ cells as a percentage of total CD8^+^ cells. Backgrounds as determined using irrelevant peptides were usually in the order of 0.1% or less and were subtracted from the values presented for test samples.

### In vitro cytotoxicity assay

Splenocytes from virus infected mice or uninfected control mice were used as effectors and peptide-loaded, CFSE-labeled RMA cells were used as targets. RMA cells (1×10^6^/ml in D0) were incubated with a 1/600 dilution of Vybrant DiD cell labeling solution (Molecular Probes) for 1 hour at 37°C, washed and split into three populations. One population was loaded with 10^−7^ M B8_20_ peptide for 1 hour at 37°C and labeled with a high concentration (2.5 µM) of CFSE for 8 min at 37°C (CFSE^high^ cells). The second population was pulsed with 10^−7^ M A3_270_ peptide and labeled with a low concentration (0.25 µM) of CFSE (CFSE^low^ cells). The third population was pulsed with 10^−7^ M of an irrelevant peptide and was left unlabelled. CFSE labeling was stopped by addition of cold D10. Cells were washed with PBS, mixed together in equal proportions and adjusted to 1×10^5^/ml D10. 100 µl (1×10^4^) of target cells were plated into V-bottom 96-well plates and co-incubated with effector splenocyte populations at effector: target (E:T) ratios of 100, 50, 20 and 10 for 16 hours at 37°C with 5% CO_2_. Cells were washed in PBS with 2% FBS and fixed in 1% paraformaldehyde before acquisition using a FACS LSR II. Analysis was done using Flowjo software. Cells were gated for live RMA cells on the FSC × SSC, followed by gating on DiD^+^ cells. DiD^+^ cells were further gated on CFSE^high^, CFSE^low^ and CFSE^neg^ cells. To calculate specific lysis, the following formula was used: ratio  =  (percentage CFSE^neg^/percentage CFSE^high^) for B8_20_ or ratio  =  (percentage CFSE^neg^/percentage CFSE^low^) for A3_270_. Percent specific lysis  =  [1– (ratio naïve effectors/ratio immune effectors) ×100].

### Activation and transfer of OT-I cells and in vivo cytotoxicity assay

Splenocytes were prepared from OT-I mice and half of them were pulsed with 10^−7^ M OVA_257_ peptide for 1 hour at 37°C. To activate OT-1 specific CD8^+^ T cells, equal numbers of pulsed and unpulsed splenocytes were co-cultured for 4 days in D10 containing 5×10^−5^ M 2-mercaptoethanol and 1.25 ng/ml rIL-2 (R&D Systems). The cultures were diluted 1/2 on days 2 and 3 with fresh medium containing rIL-2. On day 4, cells were harvested and transferred into B6 mice via intravenous (i.v.) injections in a total volume of 200 µl PBS. To prepare target cells for a CFSE-based cytotoxicity assay, naïve B6.SJL splenocytes (CD45.1^+^) were pulsed with 10^–7^ M OVA_257_ peptide for 1 hour at 37°C and labeled with a high concentration (2.5 µM) of CFSE for 8 min at 37°C (CFSE^high^ cells). Unpulsed B6.SJL splenocytes were labeled with a low concentration (0.25 µM) of CFSE (CFSE^low^ cells). CFSE labeling was stopped by addition of cold D10. Cells were washed with PBS and mixed together in equal proportions. 1–2×10^7^ mixed cells were injected i.v. into B6 mice that had been injected with OVA_257_-specific CD8^+^ T cells 24 hours earlier. Naïve mice were used as controls. Mice were sacrificed 4 hours later and spleens were harvested. CD45.1^+^ CFSE^high/low^ cells were measured to determine *in vivo* cytotoxicity. Percentage of lysis was calculated as follows: [1– (ratio naïve mouse/ratio test mouse)] ×100 where the ratio in each mouse is percentage CFSE^low^/percentage CFSE^high^.

### Statistics

A one way ANOVA was used for comparisons where there were more than two groups, with pair-wise comparisons within these data sets being done using a Bonferroni's multiple comparison post test (GraphPad Prism). Best fit linear regression and r^2^ statistics (coefficient of determination) were calculated using GraphPad Prism.
